# Development of a nomogram that predicts the risk for coronary atherosclerotic heart disease

**DOI:** 10.18632/aging.103216

**Published:** 2020-05-18

**Authors:** Shuna Huang, Xiaoxu Xie, Yi Sun, Tingxing Zhang, Yingying Cai, Xingyan Xu, Huangyuan Li, Siying Wu

**Affiliations:** 1 Department of Epidemiology and Health Statistics, School of Public Health, Fujian Medical University, Fuzhou 350122, China; 2 Department of Cardiology, The First Affiliated Hospital of Fujian Medical University, Fuzhou 350005, China; 3 Department of Preventive Medicine, School of Public Health, Fujian Medical University, Fuzhou 350122, China

**Keywords:** coronary atherosclerotic heart disease, biological, behavioral, psychological, nomogram

## Abstract

Studies seldom combine biological, behavioral and psychological factors to estimate coronary atherosclerotic heart disease (CHD) risk. Here, we evaluated the associations between these factors and CHD to develop a predictive nomogram to identify those at high risk of CHD. This case-control study included 4392 participants (1578 CHD cases and 2814 controls) in southeast China. Thirty-three biological, behavioral and psychological variables were evaluated. Following multivariate logistic regression analysis, which revealed eight risk factors associated with CHD, a predictive nomogram was developed based on a final model that included the three non-modifiable (sex, age and family history of CHD) and five modifiable (hypertension, hyperlipidemia, diabetes, recent experience of a major traumatic event, and anxiety) variables. The higher total nomogram score, the greater the CHD risk. Final model accuracy (as estimated from the area under the receiver operating characteristic curve) was 0.726 (95% confidence interval: 0.709-0.747). Validation analysis confirmed the high accuracy of the nomogram. High risk of CHD was associated with several biological, behavioral and psychological factors. We have thus developed an intuitive nomogram that could facilitate development of preliminary prevention strategies for CHD.

## INTRODUCTION

Coronary atherosclerotic heart disease (CHD) remains a major cause of global death [1, 2]. Currently in China, only about 0.2% of adults can be said to be in ideal cardiovascular health, and there are 11 million patients with CHD, which remains the second leading cause of premature death [3–5]. Although attention has been paid to alleviating the burden of CHD, efforts have focused primarily on treatment of the disease, not its prevention [6, 7]. Consequently, there is a tremendous opportunity to a shift to equal emphasis on intervention and prevention of CHD.

To formulate and optimize preventive strategies for CHD, it is essential to understand and appropriately quantify the contributions of its key risk factors. Evaluating and managing risk factors for CHD can be complex, however, as they include biological, behavioral and psychological factors [8–10]. According to Framingham study, it is estimated that over 90% of CHD events occur in persons with at least one high-risk factor (hypertension, hyperlipidemia, smoking, etc.) [11]. In addition, studies have revealed that lowering the systolic blood pressure and lipoprotein cholesterol, stopping smoking, maintaining a healthy weight, exercising, and eating a healthy diet are all associated with lower CHD risk [12–17]. Recently, the relationship between psychological stress (stressful life events, depression and demoralization) and CHD has begun to attract attention [18–21]. However, few studies have assessed the various influencing factors at the same time, making it difficult to estimate the risk of CHD poses by combinations of various types of influencing factors.

In addition, the nomogram is undergoing a resurgence as a visualization tool for disease prevention [22–24]. However, there have been few studies combining biological, behavioral, and psychological factors to establish nomograms with which to assess the risk of CHD, especially in China.

To address this knowledge gap, we performed a case-control study in southeast China. The specific goal of this study was to cover a broad spectrum of biological, behavioral and psychological factors in order to identify those that show significant associations with CHD and to clarify the comprehensive predictive value of CHD risk. The next objective, then, was to develop a nomogram with which to estimate the probability of CHD in those with risk factors.

## RESULTS

### Baseline characteristics and risk factors of CHD

Among the 4392 participants in this study, 1,578 (35.9%) suffered from CHD. The biological characteristics of cases and controls are shown in Tables 1 and 2. At baseline, patients with CHD showed were significantly older; had a higher education level; higher body mass index (BMI), waist hip ratio (WHR) and waist to height ratio (WHtR); were most likely male with family history of diabetes, CHD and stroke; and had hypertension, hyperlipidemia and/or diabetes.

**Table 1:** Baseline characteristics of the analyzed participants.

**Variables**		**Non-CHD, n(%)**	**CHD, n(%)**	**χ^2^**	*P
Sex	Female	1478(52.5)	591(37.5)	92.161	<0.001
	Male	1336(47.5)	987(62.5)		
Age	≤65	1662(59.1)	689(43.7)	96.379	<0.001
	>65	1152(40.9)	889(56.3)		
Marital status	Marriage	2529 (89.9)	1404(89)	0.873	0.350
	Single and others	285(10.1)	174(11)		
Education years	≤6	1371(48.7)	721(45.7)	7.408	0.025
	7-12	1188(42.2)	678(43.0)		
	>12	255(9.1)	179(11.3)		

*The *P* value was calculated by the Chi-square test.

Abbreviations: CHD, coronary atherosclerotic heart disease.

**Table 2:** Family history, clinical disease, and physical characteristics of the participants.

**Variables**		**Non-CHD, n(%)**	**CHD, n(%)**	**χ^2^**	*P
Family history					
Hypertension	Yes	1106(39.3)	658(41.7)	2.413	0.120
Diabetes	Yes	456(16.2)	311(19.7)	8.611	0.003
CHD	Yes	392(13.9)	315(20.0)	27.234	<0.001
Stroke	Yes	283(10.1)	212(13.4)	11.536	0.001
Clinical diseases					
Hypertension	Yes	1459 (51.8)	1188 (75.3)	231.931	<0.001
Hyperlipidemia	Yes	462 (16.4)	400 (25.3)	51.118	<0.001
Diabetes	Yes	384 (13.6)	434 (27.5)	128.095	<0.001
Physical characteristics					
Body mass index	< 18.50	138 (4.9)	48 (3.0)	19.706	<0.001
	18.50–23.99	1371 (48.7)	709 (44.9)		
	24-27.99	1026 (36.5)	626 (39.7)		
	≥ 28.00	279 (9.9)	195 (12.4)		
Waist hip ratio	Normal	576 (20.5)	262 (16.6)	9.786	0.002
	Abdominal obesity	2238 (79.5)	1316 (83.4)		
Waist to height ratio	<0.5	590 (21.0)	279 (17.6)	7.153	0.007
	≥0.5	2224 (79.0)	1300 (82.4)		

*The *P* value was calculated by the Chi-square test.

Abbreviations: CHD, coronary atherosclerotic heart disease.

The behavioral characteristics of the two groups are provided in Table 3. Smoking and a high-salt diet were related to high risk of CHD. In addition, the frequency of tea drinking and the type of edible oil consumed correlated significantly correlation with CHD.

**Table 3:** Behavioral variables of the participants.

**Variables**		**Non-CHD, n(%)**	**CHD, n(%)**	**χ^2^**	*P
Pack-year Smoking	No	1982(70.4)	954 (60.5)	66.984	<0.001
	0-20	266 (9.5)	144 (9.1)		
	20-40	367 (13.0)	275 (17.4)		
	>40	199 (7.1)	205 (13.0)		
Alcohol drinking	No	2378 (84.5)	1289 (81.7)	5.859	0.053
	<3 time/week	156 (5.5)	105 (6.7)		
	≥3 time/week	280 (10.0)	184 (11.7)		
Tea drinking	No	1790 (63.6)	944 (59.8)	8.582	0.014
	<3 time/week	282 (10.0)	153 (9.7)		
	≥3 time/week	742 (26.4)	481 (30.5)		
Physical exercise	No	952 (33.8)	527 (33.4)	4.347	0.226
	<3 time/week	393 (14.0)	194 (12.3)		
	≥3 time/week	1469 (52.2)	857 (54.3)		
Food intake	Eight full	1982 (70.4)	1069 (67.7)	3.887	0.274
	Less	233 (8.3)	151 (9.6)		
	Full	486 (17.3)	290 (18.4)		
	Not fixed	113 (4.0)	68 (4.3)		
Edible oils	Vegetable oil	2444 (86.8)	1419 (89.9)	17.537	<0.001
	Animal oil	78 (2.8)	16 (1.0)		
	Animal and vegetable	292 (10.4)	143 (9.1)		
High-salt diet	No	2003 (71.2)	1059 (67.1)	7.931	0.005
	Yes	811 (28.8)	519 (32.9)		
Vegetable	<1 day/week	30 (2.4)	21 (2.9)	4.978	0.173
	1-2 day/week	58 (4.7)	44 (6.1)		
	3-4 day/week	80 (6.5)	33 (4.6)		
	≥5 day/week	1068 (86.4)	626 (86.5)		
Fruit	<1 day/week	271 (22.0)	179 (24.8)	2.788	0.426
	1-2 day/week	341 (27.7)	200 (27.7)		
	3-4 day/week	138 (11.2)	83 (11.5)		
	≥5 day/week	481 (39.1)	259 (35.9)		
Fried food	<1 day/week	878 (75.0)	519 (74.7)	0.753	0.861
	1-2 day/week	223 (19.0)	139 (20.0)		
	3-4 day/week	48 (4.1)	27 (3.9)		
	≥5 day/week	22 (1.9)	10 (1.4)		
Fat meat	<1 day/week	814 (70.8)	483 (69.2)	6.840	0.077
	1-2 day/week	209 (18.2)	154 (22.1)		
	3-4 day/week	91 (7.9)	39 (5.6)		
	≥5 day/week	36 (3.1)	22 (3.1)		
Video duration	<1h/day	217 (21.6)	103 (17.0)	5.868	0.053
	1-3h/day	522 (52.0)	321 (53.1)		
	>3h/day	264 (26.4)	181 (29.9)		
Sleep duration	7-8h//day	480 (39.5)	262 (36.6)	3.028	0.220
	<7h//day	577 (47.5)	369 (51.5)		
	>8h//day	159 (13.1)	85 (11.9)		
Sleep quality	General	267 (21.7)	159 (22.0)	1.416	0.493
	Poor	373 (30.3)	201 (27.8)		
	Good	590 (48.0)	362 (50.1)		

*The *P* value was calculated by the Chi-square test.

Abbreviations: CHD, coronary atherosclerotic heart disease.

The psychological features of cases and controls listed in Table 4 indicate that people who have recently experienced major events and/or had depression or anxiety were prone to CHD.

**Table 4:** Psychological variables of the participants.

**Variables**		**Non-CHD, n(%)**	**CHD, n(%)**	**χ^2^**	*P
Major events encountering	No	2272 (80.7)	1226 (77.7)	5.786	0.016
	Yes	542 (19.3)	352 (22.3)		
Life satisfaction	General	889 (31.6)	482 (30.5)	2.738	0.434
	Dissatisfied	83 (2.9)	45 (2.9)		
	Satisfied	1842 (65.5)	1051 (66.6)		
Character type	A type	1045 (37.1)	621 (39.4)	0.589	0.745
	B type	1574 (55.9)	842 (53.4)		
	C type	146 (5.2)	87 (5.5)		
	D type	49 (1.7)	28 (1.8)		
Depression	No	1095 (69.9)	547 (64.3)	7.940	0.005
	Yes	472 (30.1)	304 (35.7)		
Anxiety	No	1296 (82.7)	627 (73.7)	27.608	<0.001
	Yes	271 (17.3)	224 (26.3)		

*The *P* value was calculated by the Chi-square test.

Abbreviations: CHD, coronary atherosclerotic heart disease.

### Development and validation of a CHD-predictive nomogram

**Figure 1:**
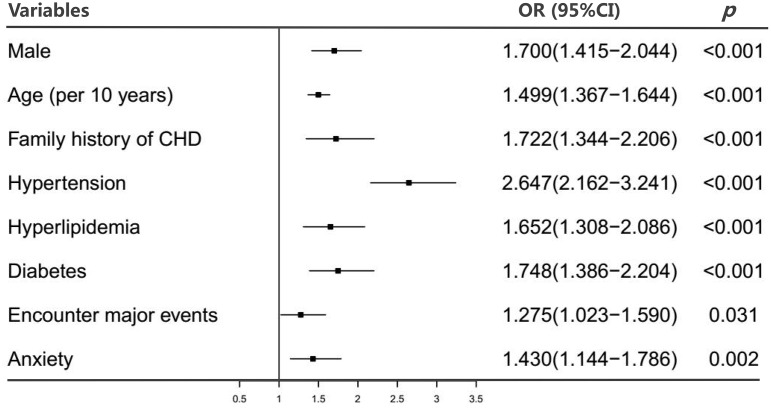
**Estimated odds ratios determined in a logistic regression model (Backwald: Wald).** Abbreviations: OR, odds ratio; CI, confidence interval.

**Figure 2:**
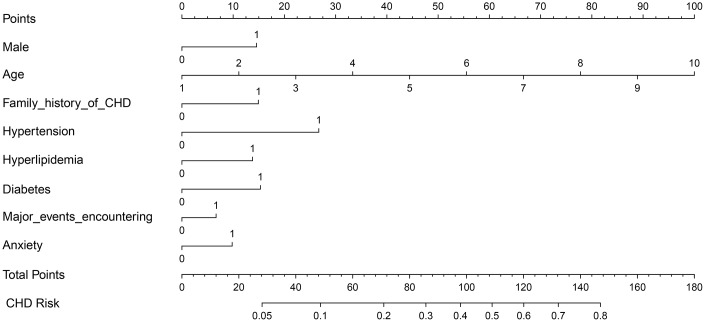
**Nomogram for predicting CHD risk.** The value of each variable was scored on a point scale from 0 to 100, after which the scores for each variable were added together. That sum is located on the total points axis, which enables us to predict the probability of CHD risk. For age categories, 1= 10 to 20, 2 = 21 to 30, 3 = 31 to 40, 4 = 41 to 50, 5 = 51 to 60, 6 = 61 to 70, 7 = 71 to 80, 8 = 81 to 90, 9 = 91 to 100, 10 = 101 to 110 year. For other variables, 0 = no and 1 = yes.

According to the “Backwald: Wald” logistic regression model, after excluding variables with *P*-values >0.05, eight predictors were associated with CHD: sex, age, family history of CHD, hypertension, hyperlipidemia, diabetes, encounters with major events, and anxiety (Figure 1). The results from logistic regression analyses were then used to construct a nomogram to predict the probability of CHD (Figure 2). To estimate the risk of CHD, the observed value of each predictor was assigned a certain number of points by drawing a vertical line towards the top points scale. The sum of the points for each variable corresponded to the individual risk of developing CHD.

**Figure 3:**
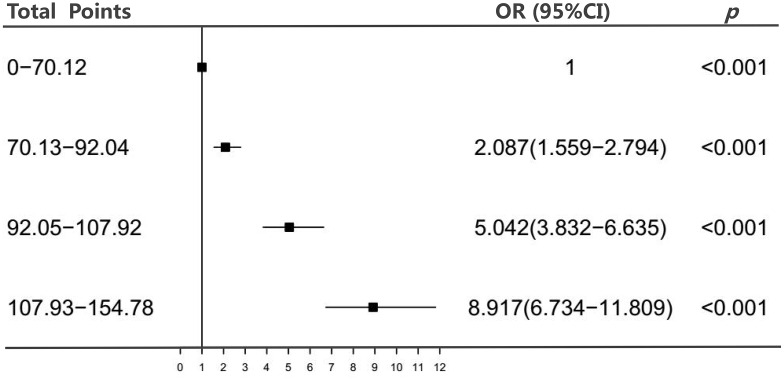
**Association between the total points of the nomogram and CHD.** Abbreviations: OR, odds ratio; CI, confidence interval.

We next divided the total points into four subgroups by quartile. Figure 3 shows that the risk of CHD increased with the total points, and participants in quartile four (total points: 107.93-154.78) had a higher CHD risk than those in the lower quartiles (odds ratio [OR]: 8.917, 95% confidence interval [CI]: 6.734-11.809).

**Figure 4:**
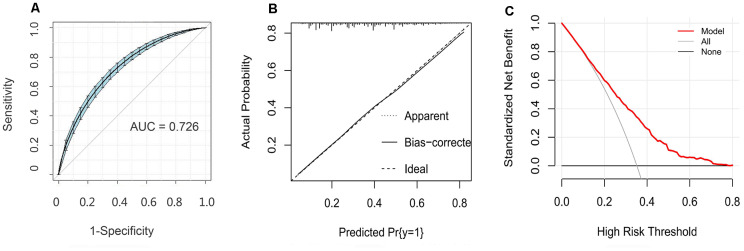
**Evaluation of the nomogram model.** (**A**) Receiver operating characteristic curve for the nomogram generated using bootstrap resampling (500 times). (**B**) Nomogram calibration plot. When the solid line (performance nomogram) was closer to the dotted line (ideal model), the prediction accuracy of the nomogram was better. (**C**) Decision curve analysis for the prediction model. The red solid line is from the prediction model, the gray line is for all patients with CHD, and the solid horizontal line indicates no patients have CHD. The graph depicts the expected net benefit per patient relative to the nomogram prediction of CHD risk. The net benefit increases as the model curve is extended.

Finally, we verified the accuracy of the nomogram. Using a bootstrap method with 500 resamples, the area under curve (AUC) for the nomogram was determined to be 0.726 (95%CI = 0.709-0.747) (Figure 4A). Among them, the AUC value of the non-modifiable variables (sex, age and family history of CHD) was 0.649 (95% CI: 0.630-0.668), and the modifiable variables (hypertension, hyperlipidemia, diabetes, encounters with major events, and anxiety) was 0.681 (95% CI: 0.662-0.700). The analysis showed that the AUC value of the modifiable variables was higher than that of the non-modifiable variables (Z = 2.053, *P* = 0.040). In addition, the probabilities predicted by the nomogram matched well with the clinical outcomes (Figure 4B), and the decision curve shows that the model has potential clinical application value (Figure 4C).

## DISCUSSION

### Key findings

In this retrospective study, our results illustrated the significant contributions of sex, age, family history of CHD, hypertension, hyperlipidemia, diabetes, recent major events, and anxiety to the risk of CHD. Using these variables, a nomogram to assess the risk of CHD was established and validated, which we suggest is of potentially great significance for the primary prevention of CHD.

### Comparison with other studies

In contrast to earlier studies, which focused on specific factors associated with CHD [11–17], we combined biological, behavioral and psychological factors, and found that the aforementioned eight variables associated with high CHD risk. Among those, were three non-modifiable factors (sex, age and family history), which is in good agreement with earlier reports [25, 26]. In addition, hypertension, dyslipidemia and diabetes are also well-established risk factors for CHD [27–29]. Our findings are consistent with the earlier findings, but extend their scope by providing data on psychological factors. We reported an OR of 1.430 (95%CI: 1.144-1.789) for relation between anxiety and CHD risk, which is consistent with previous studies [30, 31]. We also found that people who have recently experienced major events that cause traumatic stress are prone to CHD. The causal pathways between the physiological responses to psychological stress involving two regulatory axes (hypothalamic-pituitary-adrenocortical and sympatho-adrenomedullary axes) are well established, but its association with the progression of CHD remains unclear [32]. One possible mechanism by which psychological stress leads to CHD may be that the resultant imbalance between sympathetic and parasympathetic activity causes hypertension and tachycardia leading to CHD [33].

A study similar to this one also found that changes in biological, behavioral and psychosocial factors could alter the CHD five-year risk [34]. That study recruited 35- to 55-year-old office workers from 20 London-based civil service departments between 1985 to 1988. Research factors were limited to six risk factors (cholesterol level, hypertension, smoking, overweight, psychological disturbance and relationship problems), with age, sex, race, marital status and other factors as covariates. By contrast, our study was conducted in China, the participants' ages included those over 55, involved multiple occupations, included thirty-three biological, behavioral and physiological variables, and assessed these at the same time. Our study is thus a more suitable reflection of the situation of Chinese people, irrespective of their age or occupation, and can simultaneously assess the biological, behavioral and physiological factors contributing to CHD risk.

### Implications for clinicians and policymakers

A well-validated tool for predicting disease risk is important for appropriate clinical care, especially for primary prevention [35]. As part of the advances made in CHD prevention and management, the Framingham prediction algorithm has been widely used to estimate CHD risk [36, 37]. However, in some populations, including the Chinese, this algorithm would overestimate the risk of CHD [38–41]. This highlights the need to establish an effective CHD risk assessment tool in China. In addition, several recent studies used nomograms to evaluate CHD risk based on the results of various tests, including coronary computed tomography angiography, carotid ultrasound, and coronary artery calcium scoring [42–44]. These studies provided strong clinical evidence for diagnosis, but were useless for identification and evaluation of the factors that can alter CHD risk, which is more important for primary prevention. Our study, carried out in southeast China, identified five modifiable variables (hypertension, hyperlipidemia, diabetes, recent experience of major events, and anxiety) based on a logistic regression model and constructed a nomogram risk prediction model for practical application. The validation results for the nomogram show that the model has good predictive ability and clinical application value. As these factors are readily available, our risk assessment model could potentially be widely accepted.

### Strengths and limitations

A key strength of this study was our ability to simultaneously evaluate an wide range of predictors, including biological, behavioral and psychological factors. In addition, previous studies have confirmed that substantial mitigation of CHD prevention could be achieved in clinical practice through improvements in modifiable risk factors [45, 46]. We have created an easy-to-use nomogram in China that includes five modifiable variables, which are especially important for the prevention of CHD in China. Moreover, for non-modifiable variables, it is also beneficial for the public to be aware of CHD risk. That knowledge provides the public with the opportunity to voluntarily enhance early screening and promote secondary prevention. Consequently, the combination of modifiable variables and non-modifiable variables is more conducive to the prevention of CHD. Finally, our model included psychological factors, suggesting that psychological factors may not be ignored in the construction of risk models. Our research hopes to bring new hints to future model building, that is, psychological factors can be considered in constructing CHD risk assessment models based on prospective studies.

However, interpretation of these findings has three important caveats. First, like previous retrospective case-control studies, causal inference is limited. Consequently, our results and conclusions can only be used to assess the risk of CHD in the general population, and should be validated by strictly designed subsequent cohort studies. In addition, biological, behavioral and psychological factors are numerous. Although we took thirty-three variables into account, that does not cover everything. Future studies with broader variables are needed to further validate the findings of our study. Finally, this was a single-center study of only Chinese patients from a single region, which may limit its generalizability. Although our nomogram was validated using bootstraps with 500 resamples, future prospective multicenter studies are still needed to externally validate our results.

## CONCLUSIONS

This analysis shows that being male and/or elderly, having a family history of CHD, hypertension, hyperlipidemia, diabetes, recent experience of major traumatic events, and anxiety are associated with high CHD risk. We developed a user-friendly nomogram that could potentially be of benefit to the public and to policymakers in formulating effective strategies for CHD risk assessment and primary prevention.

## MATERIALS AND METHODS

### Study design and participants

The participants in this case-control study were enrolled from the Affiliated Union Hospital and the First Affiliated Hospital of Fujian Medical University (Fuzhou, China) between October 2014 and August 2019. A total of 4392 participants (1578 CHD cases and 2814 controls) confirmed their participation by signing an informed consent form. Our research program was in line with the Helsinki declaration and was approved by the review board of Fujian Medical University.

### Case-Control selection

The diagnosis of CHD was made by at least two experienced cardiologists based on coronary angiography. A patient was deemed to have CHD if at least one of the three major coronary arteries or major branches had significant coronary stenosis ≥ 50% [47]. Excluded were patients with other severe heart diseases, autoimmune diseases, diseases of other organs, or cancers. The corresponding control participants were deemed to be free of CHD after a series of evaluations, including clinical examinations and medical history assessments.

### Predictors

**Figure 5:**
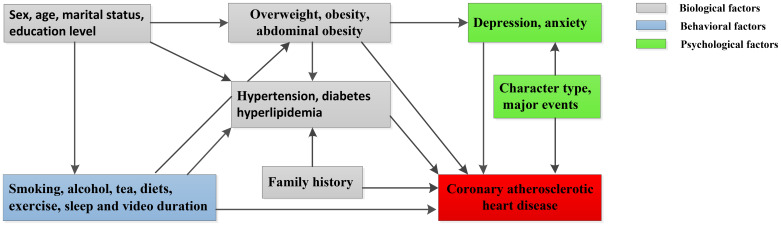
**Hypothesized association between CHD and potential predictors in our study.**

We specified the most likely temporal associations between variables based on prior biological and epidemiologic knowledge and derived predictors, including biological, behavioral and psychological factors (Figure 5). Among potential predictors, the biological variables were sex; age; marital status; educational level; family history of hypertension, diabetes, CHD or stroke; hypertension; hyperlipidemia; diabetes; BMI; WHR; and WHtR. The behavioral variables were smoking, alcohol drinking, tea drinking, diet, physical exercise, video duration, sleep duration and sleep quality. The psychological variables were major events in the last year, life satisfaction, character type, anxiety and depression. The definitions of these variables are listed in Table 5.

**Table 5:** The definition of variables in this study.

**Variables**	**Definition**
Body mass index (BMI)	BMI = weight (kg) / [height (m)]^2^. <18.50 kg/m^2^ (underweight), 18.5–23.99 kg/m^2^ (normal), 24-27.99 kg/m^2^ (overweight), ≥28.00 kg/m^2^ (obese) [48].
Waist hip ratio (WHR)	WHR = waist / hip. Abdominal obesity: ≥0.85 (women); ≥0.90 (men) [49].
Waist to height ratio (WHtR)	WHtR= waist (m) / height (m), and WHtR ≥0.5 was defined as obesity [50].
Pack- year Smoking	Pack- year Smoking= (No. of years of smoking _*_ average no. of cigarette smoked per day) / 20 cigarettes in a pack [51].
Alcohol drinking	Alcohol drinkers were defined according to literature [52], and divided into the following three levels: no drinking, <3 time/week and ≥3 time/week.
High-salt diet	Daily salt intake was calculated by averaging a family's annual salt consumption with the number of members [53], and a high-salt diet means eating more than 6 grams of salt per day.
Food	Vegetable, fruit, fried food and fat meat eaten 1 or more times a day were recorded as 1 day, and the weekly vegetable, fruit, fried food and fat meat of all subjects were determined.
Physical exercise	Effective physical activity referred to exercise that lasts for least 20 minutes each time, and was used to define weekly physical activity for all subjects.
Sleep quality	Sleep quality was defined by asking whether the respondents had difficulty falling asleep or/and staying asleep during the past year. They were divided into 5 levels: very good (<1 day/month), good (1-3 days/month), general (4-7 days/month), poor (8-15 days/month) and every poor (≥16 days/month or need to take medicine to sleep), which are filled out by the respondents themselves.
Encountering major events	Encountering major events referred to accidents such as death or serious illness of family members, family bankruptcy, unemployment, marital barriers in the past year.
Anxiety	Anxiety was evaluated by self-rating anxiety scale (SAS). SAS scores ≥50 was used to indicate anxiety, and the Cronbach’s α in this study was 0.85 [54].
Depression	A self-rating depression scale (SDS) was performed to estimate depression. SDS ≥53 was defined as depression, and the Cronbach’s α in this study was 0.86 [54].

### Statistical analysis

The characteristic differences between the case and control groups were evaluated using the Chi-square test. Variables with *P*-values <0.05 in the univariate analyses were introduced as independent predictors into a multivariate logistic regression. We estimated the strength of the association between CHD risk and predictors by OR and 95% CI. Significant variables were selected using the “Backwald: Wald” method and were used to construct a nomogram. The total score of the nomogram was classified by quartile to assess the association of the total score with CHD risk. The discriminative ability, predictive accuracy and clinical application value of the model was assessed using a receiver operating characteristic (ROC) curve, calibration plot, and decision curve analysis. A total of 500 bootstrap resamples were employed to reduce over-fit bias. Statistical analyses were performed using SPSS version 25.0, R version 3.5, MedCalc and EmpowerStats for Windows. Two-sided *P* values < 0.05 was considered significant.
